# Dispatch from the field II: the mystery of the red and blue *Opadometa* male (Araneae, Tetragnathidae, *Opadometa sarawakensis*)

**DOI:** 10.3897/BDJ.6.e24777

**Published:** 2018-04-03

**Authors:** Jeremy A. Miller, Christian Freund, Liselotte Rambonnet, Lianne Koets, Nadine Barth, Corné van der Linden, József Geml, Menno Schilthuizen, Richard Burger, Benoit Goossens

**Affiliations:** 1 Naturalis Biodiversity Center, Leiden, Netherlands; 2 Plazi.org, Bern, Switzerland; 3 Leiden University Medical Center, Leiden, Netherlands; 4 Leiden University, Leiden, Netherlands; 5 Wageningen University, Wageningen, Netherlands; 6 Taxon Expeditions, Leiden, Netherlands; 7 Universiti Malaysia, Kota Kinabalu, Malaysia; 8 Danau Girang Field Centre, Sabah, Malaysia; 9 Cardiff School of Biosciences, Cardiff University, Cardiff, United Kingdom; 10 Sustainable Places Research Institute, Cardiff University, Cardiff, United Kingdom; 11 Sabah Wildlife Department, Sabah, Malaysia

**Keywords:** Borneo, tropical field course, spider, orb web, sexual size dimorphism

## Abstract

**Background:**

Males of *Opadometa* are difficult to associate with conspecific females, and sex-matching errors may persist in the taxonomic literature. Recommended best practices for definitive sex matching in this genus suggest finding a male in the web of a female, or better yet, mating pairs.

**New information:**

A male *Opadometa* was observed hanging on a frame line of the web of a female *Opadometa sarawakensis*, a species for which the male was previously undescribed. This occurred during a tropical ecology field course held at the Danau Girang Field Centre in Sabah, Malaysia. A taxonomic description was completed as a course activity.

## Introduction

The cover of Koh and Ming’s 2014 field guide to the Spiders of Borneo was graced with a striking but at the time undescribed red and blue *Opadometa* species. Koh and Ming (2014) included a discussion on the “Mysteries of the *Opadometa* Males”, in which they detailed the complexities of associating the rarely collected males of *Opadometa* with conspecific females. They included photographs of a male *Opadometa* collected in the vicinity of the red and blue female, but warned that it would be premature to conclude that these are conspecific. Males of *Opadometa* are rare in collections and notoriously difficult to associate with conspecific females, which are more than twice their length and much heavier. Confirmation of male-female conspecificity, they state, should be accepted, “…only if the males and females are found in the same web, or better still, are seen copulating.” (p. 260).

Dzulhelmi et al. (2015) published a formal description of what is almost certainly the same red and blue *Opadometa* species based on a female specimen from Sarawak, Malaysia. The male was not described, and Dzulhelime et al. echoed some of the sentiment expressed by Koh and Ming regarding the difficulties of associating male and female *Opadometa*.

In 2018, students participating in a two-week tropical ecology field course offered by the Naturalis Biodiversity Center and Leiden University and hosted by the Danau Girang Field Centre (DGFC) in Sabah, Malaysia, found a mature male at the margin of the web of a red and blue *Opadometa*. The female spider matched Dzulhelmi et al.’s (2015) description of *Opadometa sarawakensis*. A survey of orb web-building spiders near DGFC found no other *Opadometa* species. The students resolved, along with lecturers and field station scientific staff, to describe the male and provide additional data on the female, as well as data on the ecology and behavior of the species, and submit their results in the form of a manuscript before the end of the course. This is the second contribution to spider taxonomy and natural history to be produced in this way (Miller et al. 2014).

### Males of *Opadometa*

**Figure 1.**
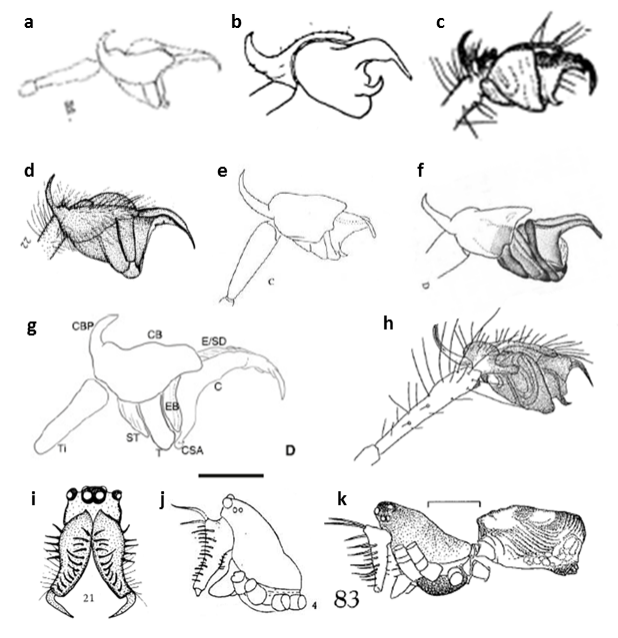
Selected images from the legacy taxonomic literature depicting the male of purported *Opadometa grata* (according to the current World Spider Catalog 2018) with notation of geographic location. **a-h**: pedipalp with attention to the cymbial basal apophysis (CBP, see Kuntner et al. 2008). **i-k**, male prosoma with attention to the chelicerae. **a**: Kulczyński 1911: plate 19, fig. 31 from New Guinea. **b**: Berland 1938: fig. 117 (reversed), from New Hebredes. **c**: Archer 1951: fig. 13 (reversed), location unspecified (New Guinea?). **d**: Chrysanthus 1963: fig 22, New Guinea. **e**: Wang 1991: fig. 6, China (reproduced as fig. 123C in Song et al. 1999). **f**: Zhu et al. 2003: fig. 161D, China. **g**: Kuntner et al. 2008: fig. 18D, New Guinea. **h**: Ono 2011: fig. 87 (reversed), Japan. **i**: Chrysanthus 1963: fig 21, New Guinea. **j**: Wang 1991: fig. 4, China. **k**: Ono 2011: fig. 83, Japan.

Four species (plus eight subspecies) of *Opadometa* are currently cataloged (World Spider Catalog 2018): *O. grata* (Guérin, 1838), *O. fastigata* (Simon, 1877), *O. kuchingensis* Dzulhelmi & Suriyanti, 2015, and *O. sarawakensis* Dzulhelmi & Suriyanti, 2015 (World Spider Catalog 2018). The type species is *O. grata*, described from New Guinea. Males have been described and illustrated multiple times under this name (Kulczyński 1911, Berland 1938, Archer 1951, Chrysanthus 1963, Wang 1991, Song et al. 1999, Zhu et al. 2003, Kuntner et al. 2008, Ono 2011; Fig. 1). *Opadometa fastigata* was originally described from the Philippines. The male was described (as *Callinethis elegans* Thorell, 1895 from Burma, synonymized with *fastigata* by Pocock 1900) in a lengthy Latin text but has never been illustrated. The eight subspecies (seven of *O. grata* and one of *O. fastigata*) were established on the order of 100 years ago and have not been revisited (Strand 1911, Strand 1915, Hogg 1919); their status seems dubious, but this issue is outside the scope of this contribution. The two species recently described in Dzulhelmi et al. (2015) were based on females only.

Some authors have expressed skepticism that males and females of the two classical species are properly matched (e.g., Murphy and Murphy 2000, Koh and Ming 2014) because males fitting descriptions of *O. grata* have been collected together with females of *O. fastigata* (e.g., in Singapore). Examination of illustrations of the male pedipalp in the legacy taxonomic literature reveals a clue: the Cymbial Basal Process (CBP) of specimens from New Guinea and New Hebrides extend more or less retrolaterally from the cymbium before curving distally (*Fig. 1*a, b, c, d, g); males from elsewhere have the CBP extend nearly posteriorly before curving (Fig. 1e, f, h). So it could be that records of *O. grata* west of New Guinea are erroneous, and the widespread Southeast Asian and tropical East Asian species is *O. fastigata*.

## Materials and methods

Trails around the Danau Girang Field Center were surveyed for *Opadometa* and other medium to large orb weaving spiders during day and night searches. Webs were assessed for several characteristics: diameter, angle, number of radii, number of spirals, and height of hub from the ground. Vegetation density was assessed by counting stems at breast height in a circle around the web hub with a radius of 3 meters. Percent canopy cover above the hub was estimated using a photograph processed with ImageJ (Rasband 2016).

### Laboratory methods

**Figure 2.**
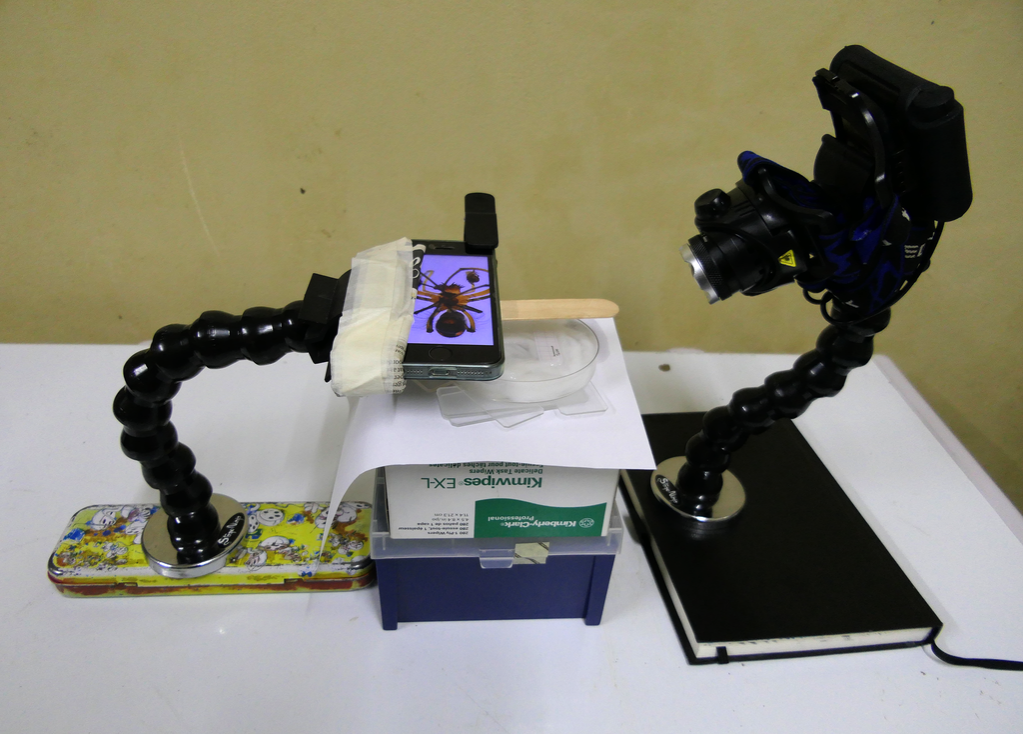
Improvised specimen imaging station at Danau Girang Field Centre.

Specimens were fixed and stored in 70% denatured ethanol. For imaging, specimens were positioned using cotton wool in a petri dish. Images were taken with an iPhone SE illuminated with an LED head light mounted on a gooseneck clip. The iPhone was mounted on a second gooseneck clip with a magnetic base and stabilized with newspaper, tape and a tongue depressor (Fig. 2). Some images were taken using a macro lens clip added to the iPhone camera. Versions of some images were taken with 5 mm graph paper as a guide for creating scale bars. Measurements are in millimeters unless otherwise specified. Color and levels were adjusted using Photoshop CS6. Anatomical abbreviations in text and figures follow Kuntner et al. (2008). All specimens have been deposited at the Universiti Malaysia Sabah's Institute for Tropical Biology and Conservation, Borneensis (BORN).

### Legacy taxonomic literature

The taxonomic publication featuring the original treatment of *Opadometa sarawakensis* was semantically enhanced with XML markup using GoldenGATE Imagine (Sautter 2018) software and deposited at Plazi.org's TreatmentBank (see also Miller et al. 2015).

## Taxon treatments

### Opadometa sarawakensis

Dzulhelmi & Suriyanti, 2015

Opadometa sp. in Koh and Ming 2014: p. 259-260.
Opadometa sarawakensis Dzulhelmi & Suriyanti, in Dzulhelmi et al. 2015: p. 102-103, figs 5-13. (Link to treatment on Plazi)

#### Materials

- **Type status:** Other material. **Occurrence:** catalogNumber: DGFCW2018022300; recordedBy: Jeremy Miller and Christian Freund; individualCount: 2; sex: 1 male, 1 female; lifeStage: adult; **Taxon:** scientificName: Opadometa sarawakensis Dzulhelmi & Suriyanti, 2015; **Location:** country: Malaysia; stateProvince: Sabah; locality: Danau Girang Field Centre trails; verbatimElevation: 23 m; decimalLatitude: 5.41619; decimalLongitude: 118.0426; **Event:** eventDate: 2018-02-23; **Record Level:** institutionID: Universiti Malaysia Sabah; collectionID: Institute for Tropical Biology and Conservation, Borneensis; institutionCode: UMS; collectionCode: BORN; basisOfRecord: PreservedSpecimen
- **Type status:** Other material. **Occurrence:** catalogNumber: DGFCW2018022402; recordedBy: Jeremy Miller, Christian Freund, Liselotte Rambonnet, Lianne Koets, Natasha Zulaikha, and Jozsef Geml; individualCount: 1; sex: female; lifeStage: adult; **Taxon:** scientificName: Opadometa sarawakensis Dzulhelmi & Suriyanti, 2015; **Location:** country: Malaysia; stateProvince: Sabah; locality: Danau Girang Field Centre trails; verbatimElevation: 23 m; decimalLatitude: 5.40999; decimalLongitude: 118.04204; **Event:** eventDate: 2018-02-24; **Record Level:** institutionID: Universiti Malaysia Sabah; collectionID: Institute for Tropical Biology and Conservation, Borneensis; institutionCode: UMS; collectionCode: BORN; basisOfRecord: PreservedSpecimen
- **Type status:** Other material. **Occurrence:** catalogNumber: DGFCW2018022611; recordedBy: Jeremy Miller and Christian Freund; individualCount: 1; sex: female; lifeStage: adult; **Taxon:** scientificName: Opadometa sarawakensis Dzulhelmi & Suriyanti, 2015; **Location:** country: Malaysia; stateProvince: Sabah; locality: Danau Girang Field Centre trails; verbatimElevation: 22 m; decimalLatitude: 5.41623; decimalLongitude: 118.04273; **Event:** eventDate: 2018-02-26; **Record Level:** institutionID: Universiti Malaysia Sabah; collectionID: Institute for Tropical Biology and Conservation, Borneensis; institutionCode: UMS; collectionCode: BORN; basisOfRecord: PreservedSpecimen

#### Description

Male: from Sabah, Malaysia (DGFCW2018022300). Prosoma uniform orange. Eight eyes in two rows, with the medians closer together than to the laterals; posterior median eyes oriented slightly toward the front; lateral eyes touching. Sternum dusky orange, darker posteriorly. Chelicerae orange, enlarged, diverging distally, armed in front and basolaterally with strong macrosetae; macrosetae absent from frontal-basal region (Fig. 3); with 2 promarginal and 2 smaller retromarginal teeth. Legs without macrosetae; femur of legs I and IV orange, distal segments darker; legs II and III overall dark (Fig. 4a); femur IV with row of very long prolateral trichobothria along entire length (Fig. 3a, b).

Abdomen gray dorsally with silvery patches and an anteriodorsal dark spot, black posteriorly and ventrally with two posteriolateral and one ventral orange spot, with a small anteriolateral black spot and a larger posteriolateral black spot, which joins with the black ventral marking.

Palpal trochanter, femur, and tibia very long (Fig. 3c). Cymbial basal process projects posteriorly, tip curved retrolaterally. Paracymbiuim (P) thick basally with curved distal projection. Tegulum (T) somewhat bulbous. Conductor (C) envelops embolus, thick basally, tapering through spiral path, with basal secondary apophysis (CSA) (Fig. 5).

**Figure 7.**
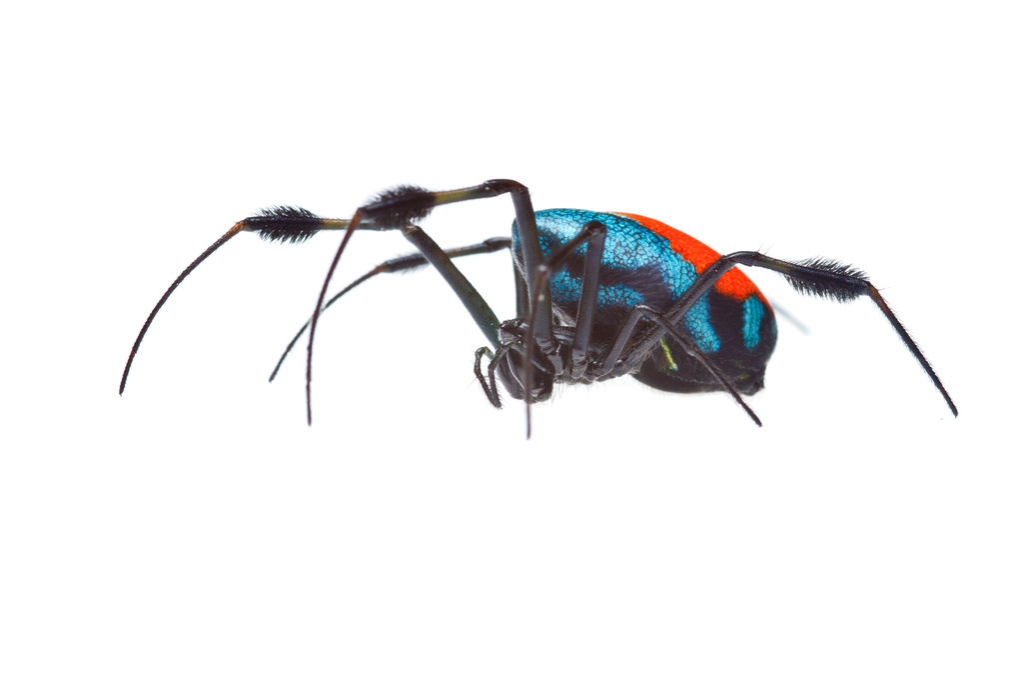
Female *Opadometa sarawakensis* Dzulhelmi & Suriyanti, 2015 (DGFCW2018022402), live habitus.

Female: For description and diagnosis of female, see Dzulhelmi et al. (2015) (also available on Plazi’s treatmentBank here). Our observations of females from DGFC largely agree with the description in Dzulhelmi et al. (2015) (Figs 4b, 6, 7). We would only add that we observed a light distal brush on tibia II in addition to the heavy brushes found on tibiae I and IV (Figs 4b, 6).

##### Measurements

Male (DGFCW2018022300): Total length 2.8; carapace length 1.4, width 0.9; abdomen length 1.4, width 0.9, height 0.9.

Female (DGFCW2018022300): Total length 6.2; carapace length 3.6, width 2.4; abdomen length 5.0, width 2.8, height 2.7.

Female (DGFCW2018022402): Total length 8.1; carapace length 3.4, width 2.7; abdomen length 7.6, width 4.2, height 4.2.

Female (DGFCW2018022611): Total length 5.0; carapace length 3.5, width 2.1; abdomen length 4.4, width 2.6, height 2.6.

#### Diagnosis

Cymbial basal process (CBP) of male palp projects initially posteriorly (Fig. 5c), distinguishing it from males illustrated from New Guinea and New Hebrides (presumably true *O. grata; Fig. 1*a, b, c, d, g) in which the CBP extends almost retrolaterally from the cymbium before curving distally; distinguished from males illustrated from further West in Southeast Asia (presumably true *O. fastigata*) by the length of the CBP, which is shorter and more gradually curved in *O. sarawakensis* (Fig. 5c) than in *O. fastigata* (Fig. 1e, f, h). *Opadometa* males further distinguished by the base of the chelicerae, which project forward with large distally oriented macrosetae in *O. fastigata* (Fig. 1j, k); absent in the male of *O. sarawakensis* (Fig. 3c, d), which is naked at the base of the chelicerae, and also apparently absent in *O. grata*, although the lateral view of male *Opadometa* from New Guinea and points East has never been illustrated (but see Fig. 1i). The male chelicerae of *O. sarawakensis* (Fig. 3d) and *O. grata* (Fig. 1i) are divergent distally, not divergent in *O. fastigata*. Males of *O. sarawakensis* may be further distinguished from those of *O. fastigata* by the orientation of the posterior median eyes (Fig. 3c), which are set further back in *O. fastigata* and are oriented slightly posteriorly in lateral view (Fig. 1k). The secondary conductor apophysis (SCA) in *O. sarawakensis* (Fig. 5a, b, d) appears to be shorter than in either *O. grata* (Fig. 1b, c, g) or *O. fastigata* (Fig. 1e, h). At 2.8, the total length of the male of *O. sarawakensis* appears to be intermediate between *O. fastigata* (1.86-2.06; Ono 2011) and *O. grata* (3.2-3.5; Kulczyński 1911, Chrysanthus 1963).

#### Distribution

*Opadometa sarawakensis* is known from lowland dipterocarp forest in Bako National Park, Sarawak and Maliau Basin, Sabah, Malaysia (Dzulhelmi et al. 2015), Danau Girang Field Centre, Sabah, Malaysia, and wooded areas and disturbed forest in Brunei (Koh and Ming 2014).

#### Ecology

Trails around the Danau Girang Field Center were surveyed for *Opadometa* and other medium to large orb weaving spiders during day and night searches. *Opadometa sarawakensis* was the largest orb-weaver encountered after *Nephila* (2 juveniles in the 10-12 mm size range; no adult *Nephila* were encountered during the survey, although they have been seen at other times); no other *Opadometa* species were encountered. *Opadometa sarawakensis* build open-hub webs with an inflection point so that the top half is more steeply inclined than the bottom half. The specific angles were quite different between the two webs measured (Fig. 8, Table 1). Webs are moderately open with relatively few radii and spirals, especially compared to the dense webs of *Nephila*. All *Opadometa* we encountered were found during the day with spiders either at the hub or web margin; night searches failed to discover any additional *Opadometa*. Despite dedicated searches over the few days and nights available, *Opadometa* seem to be quite rare in the forest of Danau Girang in February 2018. The Danau Girang Field Center is located within the Lower Kinabatangan Wildlife Sanctuary, a narrow network of lowland riparian and riverine forest patches and corridors along the Kinabatangan River, surrounded by oil palm plantations.

#### Sexual size dimorphism

Sexual size dimorphism in *Opadometa* is extreme. The male-female pair found together (DGFCW2018022300) have a female/male size ratio of 2.2, meaning the female is more than twice the total length of the male.

## Discussion

Given the troubled history of matching sexes in *Opadometa*, Koh and Ming (2014) were justifiably conservative in not prematurely concluding that the male *Opadometa* they found in proximity to the red and blue *Opadometa* was conspecific. In light of the new data presented here, it appears that the male photographed in Koh and Ming (p. 260) is in fact *O. sarawakensis*.

The evidence for matching sexes in *O. sarawakensis* presented here is behavioral (male at margin of female web) and faunistic (only one *Opadometa* species found in survey of orbweaving spiders). Another possible line of evidence would be DNA barcode sequences (Hebert et al. 2003, Barrett and Hebert 2005), which have been used to establish male-female conspecificity in sexually dimorphic spiders (Barone et al. 2016, Magalhaes et al. 2017). For an unplanned field discovery such as this one, arranging for sequencing at a domestic institution requires time and resources beyond the scope of this study. Technologies for DNA barcoding in the field appear tantalizingly close to practicality and one of us (MS with Taxon Expeditions) is involved with field trials of the MinION, a portable DNA sequencer from Oxford Nanopore Technologies (Menegon et al. 2017). We hope to see more successful DNA sequencing under field conditions in the near future, but for now, we will have to be content with non-molecular (e.g., behavioral and faunistic) lines of evidence for sex matching in remote field studies.

More work clearly needs to be done to sort out the distributions of the known *Opadometa* species. If our analysis based on the legacy of male descriptions is correct, and *O. grata* is limited to New Guinea (and possibly points East) while *O. fastigata* is found further West, this should clear up some of the confusion regarding the distribution, sex matching, and anatomical features found in this genus.

## Supplementary Material

XML Treatment for Opadometa sarawakensis

## Figures and Tables

**Figure 3a.**
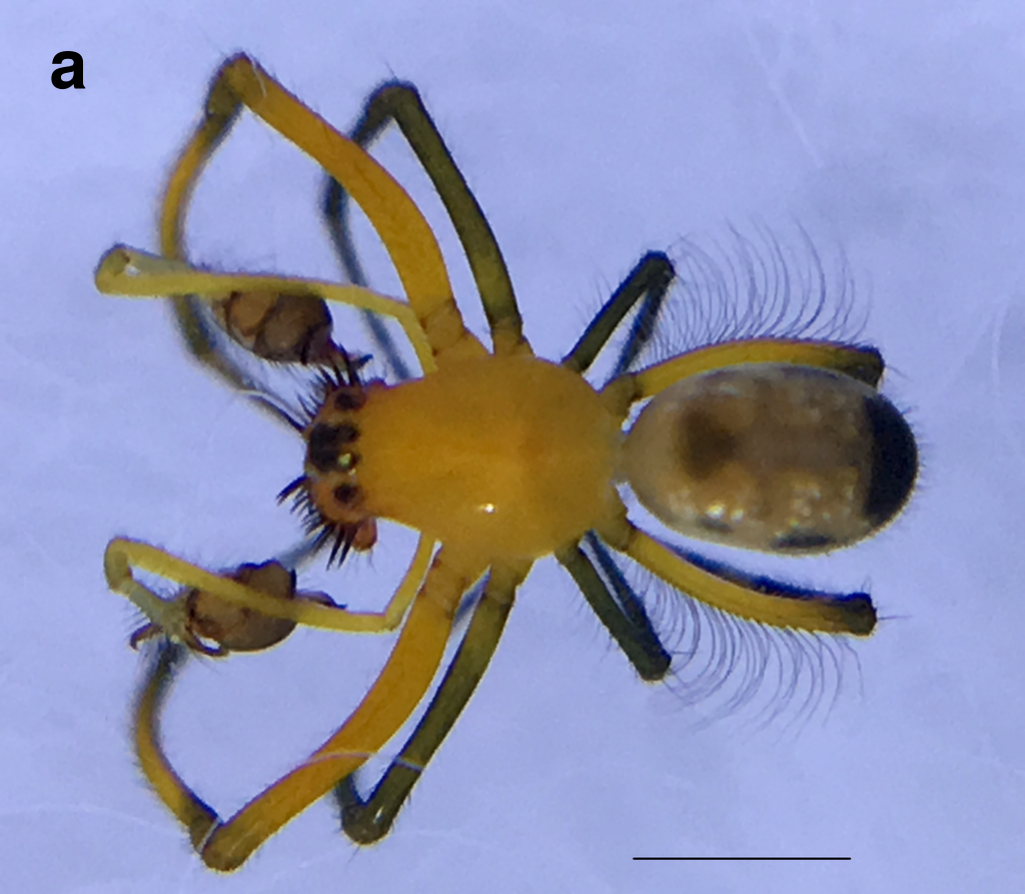
Dorsal view. Scale bar 1 mm.

**Figure 3b.**
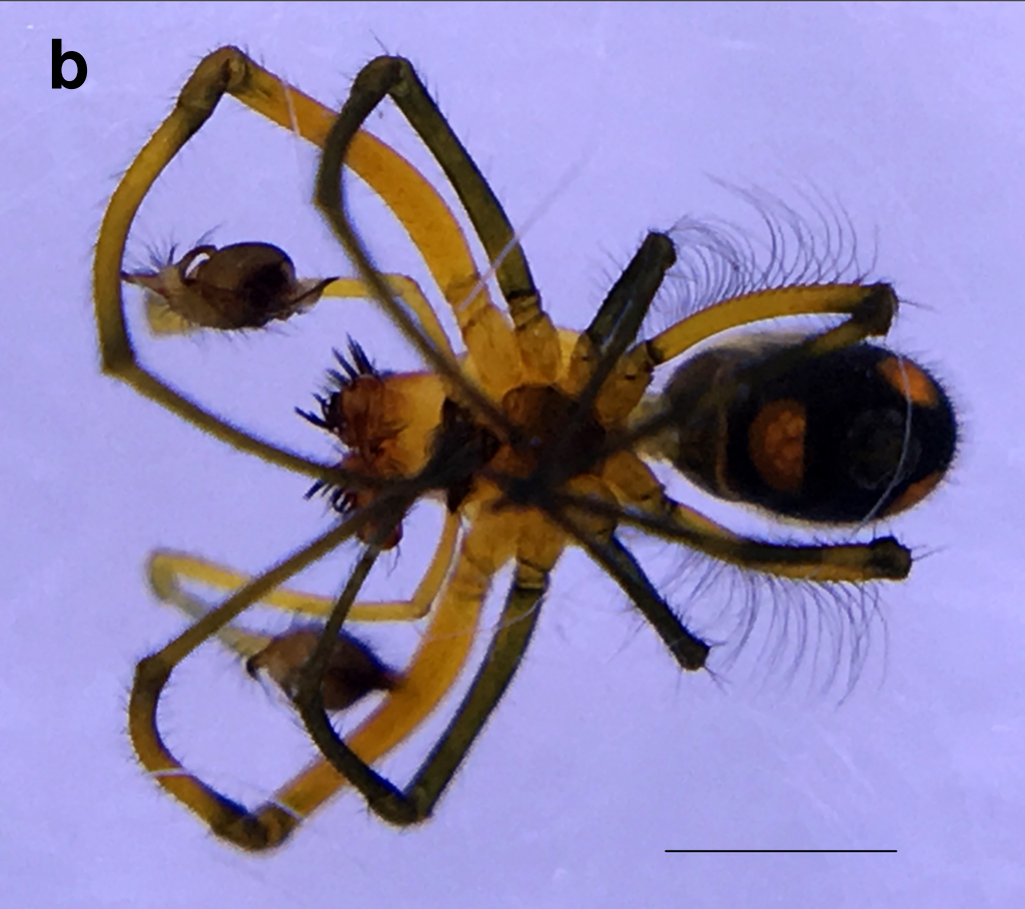
Ventral view. Scale bar 1 mm.

**Figure 3c.**
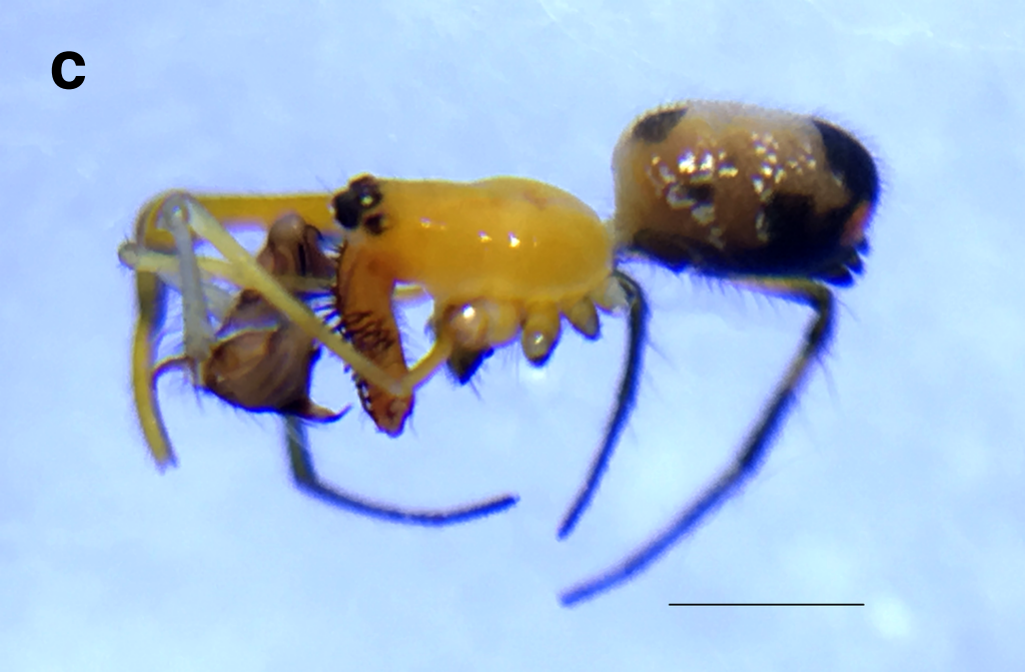
Left lateral view. Scale bar 1 mm.

**Figure 3d.**
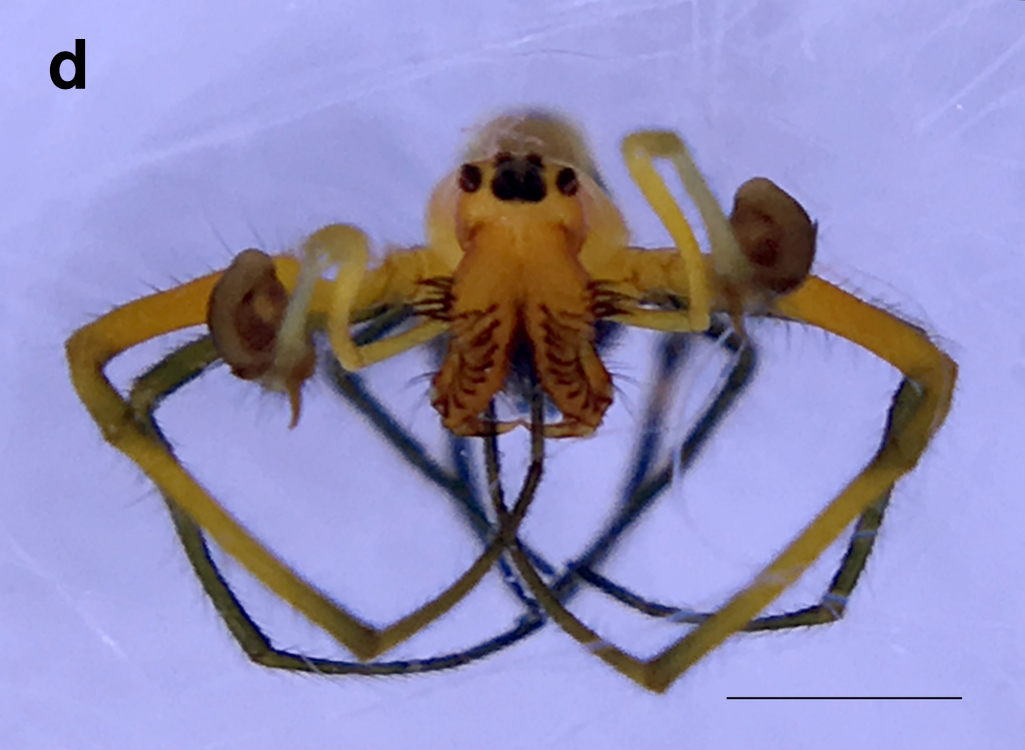
Frontal view. Scale bar 1 mm.

**Figure 4a.**
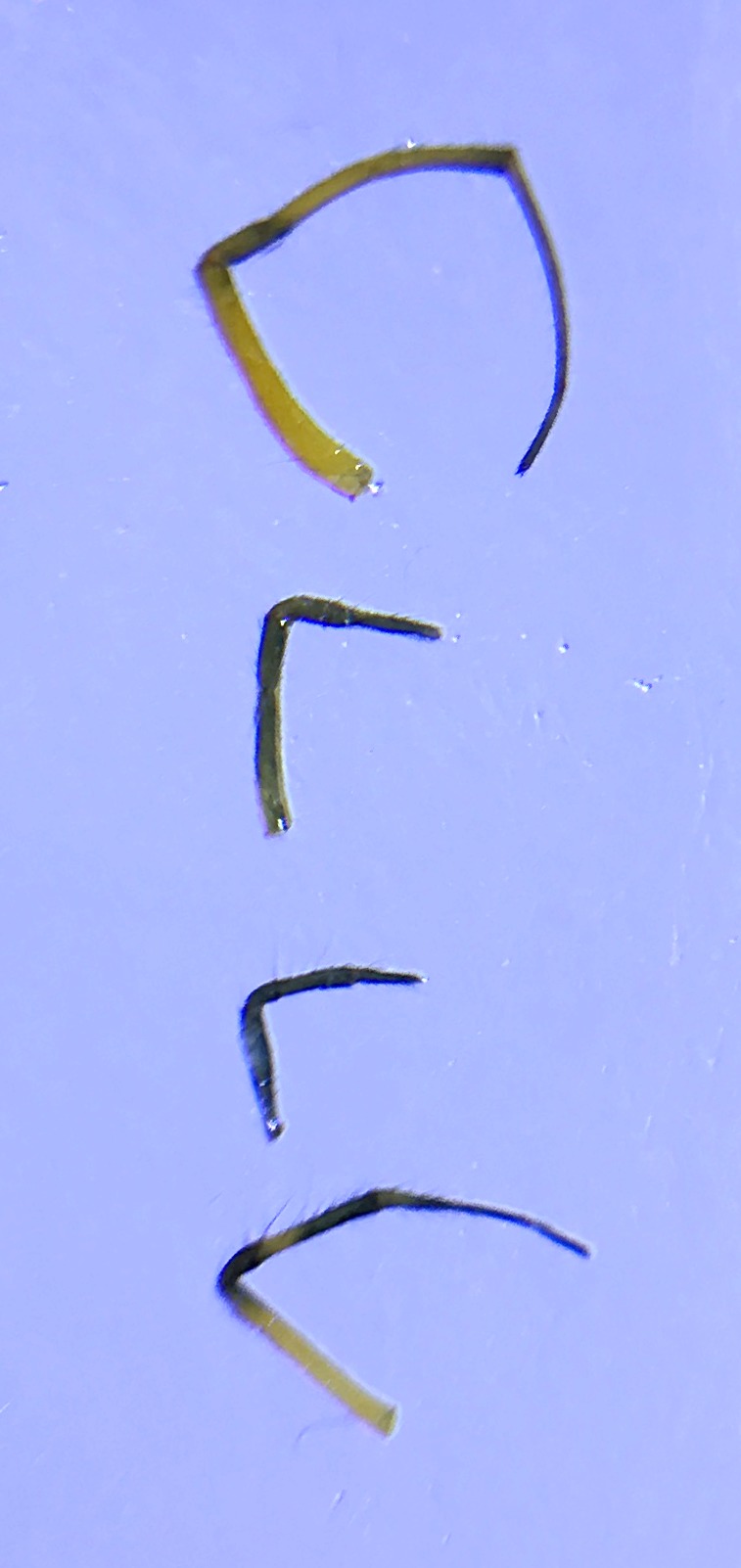
Left legs of male (DGFCW2018022300), arranged top to bottom I-II-III-IV. Leg II broken, missing metatarsus and tarsus.

**Figure 4b.**
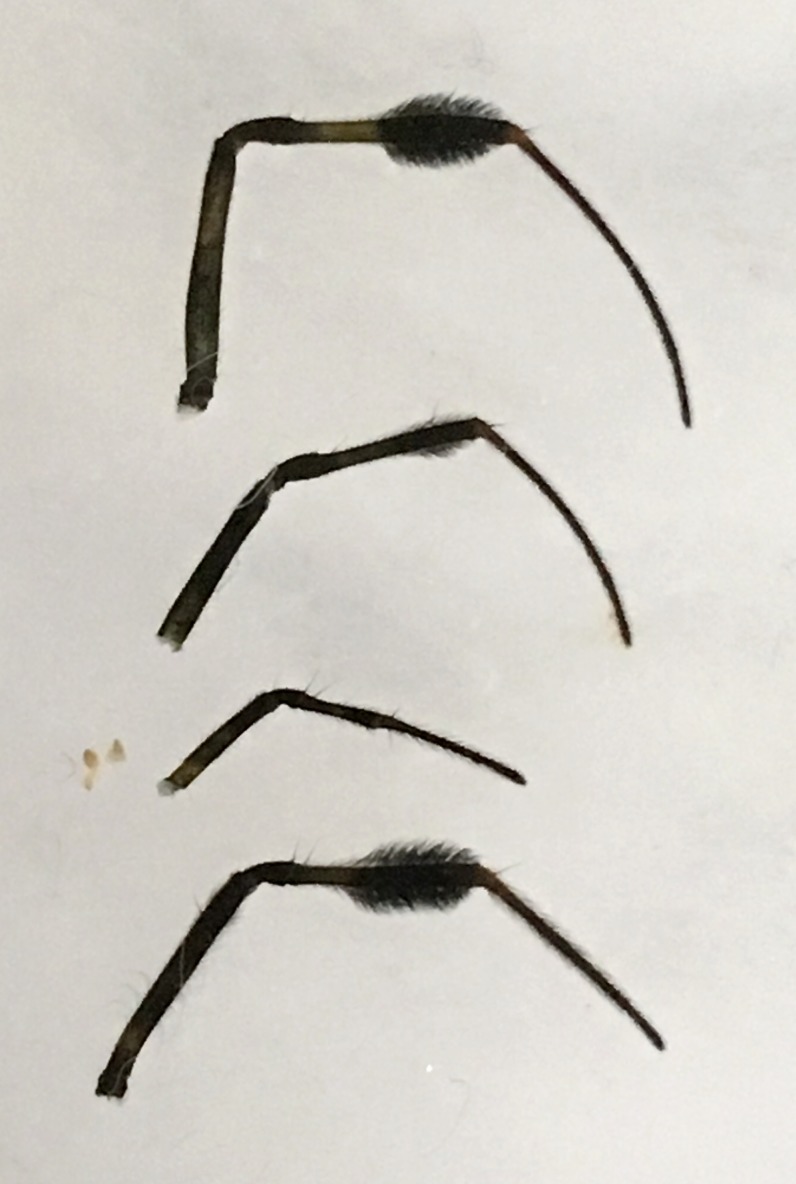
Left legs of female (DGFCW2018022402), arranged top to bottom I-II-III-IV. Note large tufts of setae on distal part of tibiae I and IV, and more diminutive tuft on tibia II.

**Figure 5a.**
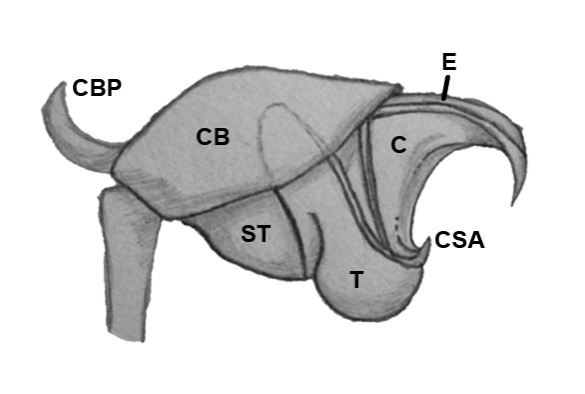
Prolateral view. C, conductor; CB, cymbium; CBP, cymbial basal apophysis; CSA, conductor secondary apophysis; E, embolus; ST, subtegulum; T, tegulum.

**Figure 5b.**
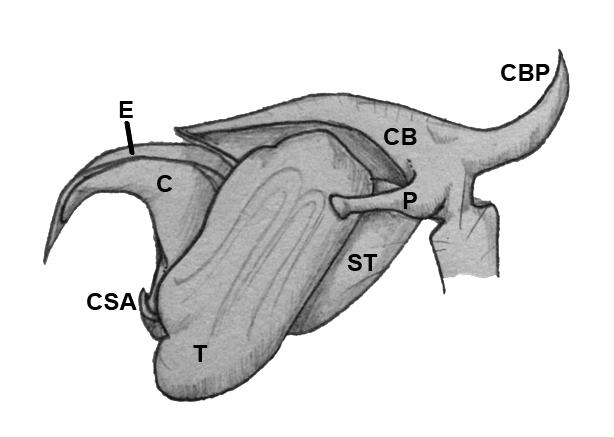
Retrolateral view. C, conductor; CB, cymbium; CBP, cymbial basal apophysis; CSA, conductor secondary apophysis; E, embolus; P, paracymbium; ST, subtegulum; T, tegulum.

**Figure 5c.**
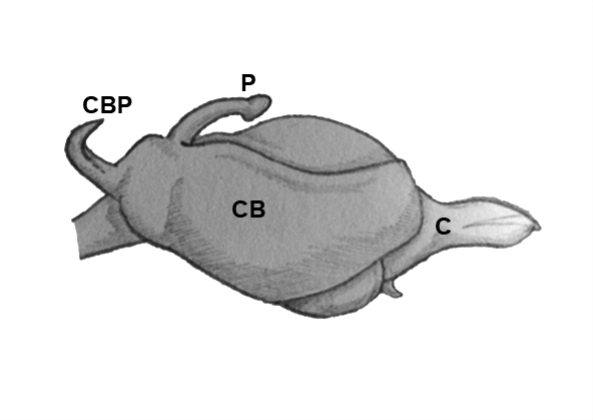
Dorsal view. C, conductor; CB, cymbium; CBP, cymbial basal apophysis; P, paracymbium.

**Figure 5d.**
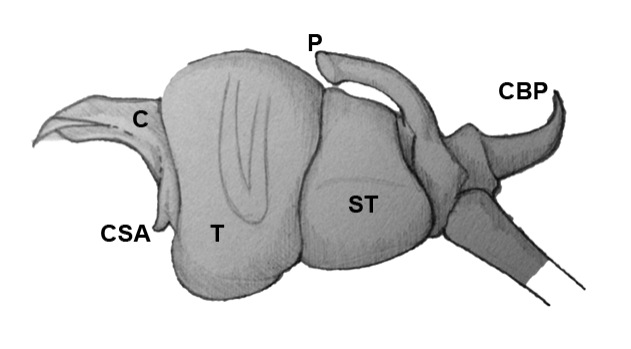
Ventral view. C, conductor; CSA, conductor secondary apophysis; P, paracymbium; ST, subtegulum; T, tegulum.

**Figure 6a.**
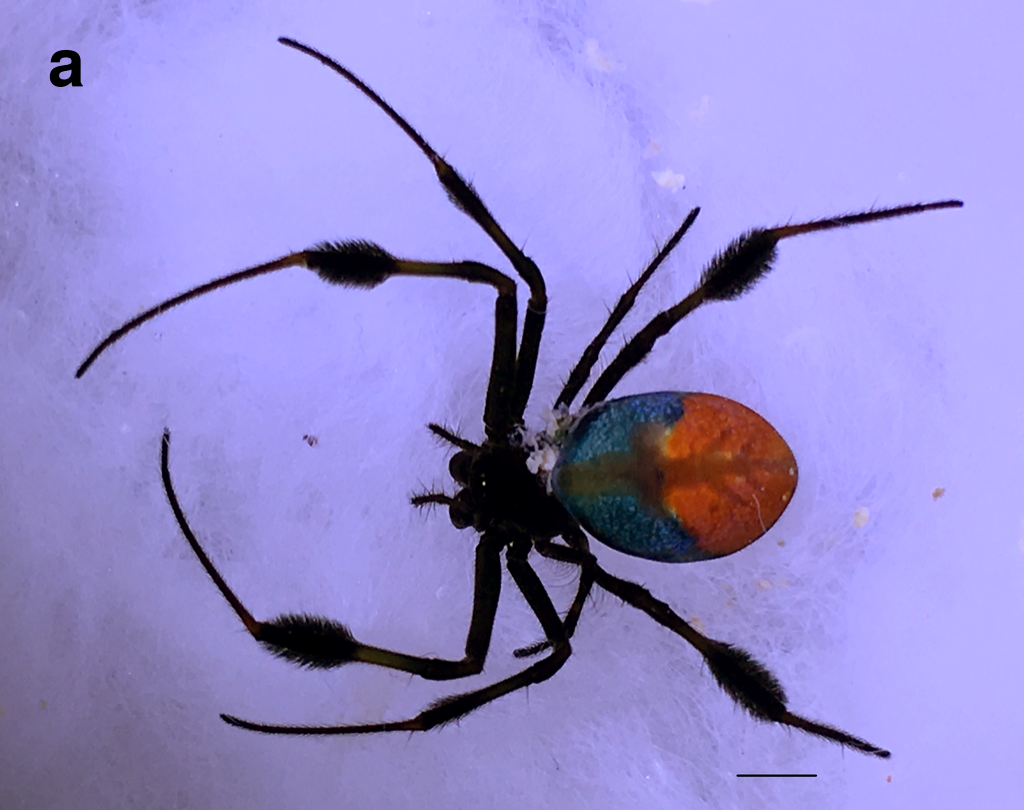
Female, dorsal view. Scale bar 2 mm.

**Figure 6b.**
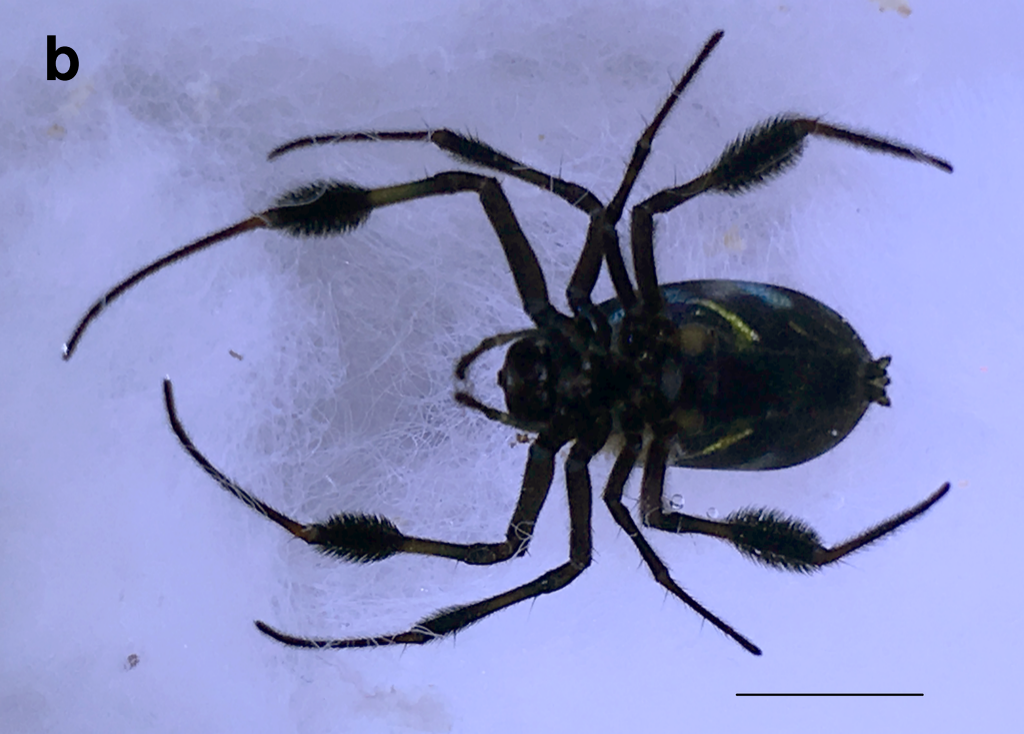
Female, ventral view. Scale bar 2 mm.

**Figure 6c.**
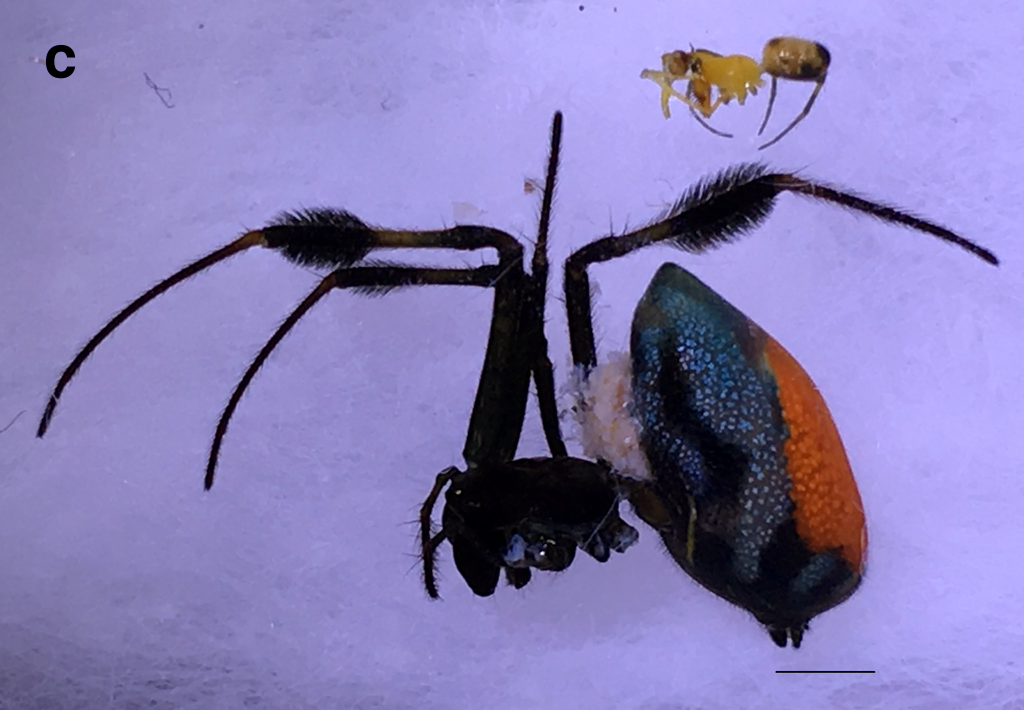
Female, lateral view, with male (DGFCW2018022300) to show sexual size dimorphism. Scale bar 2 mm.

**Figure 6d.**
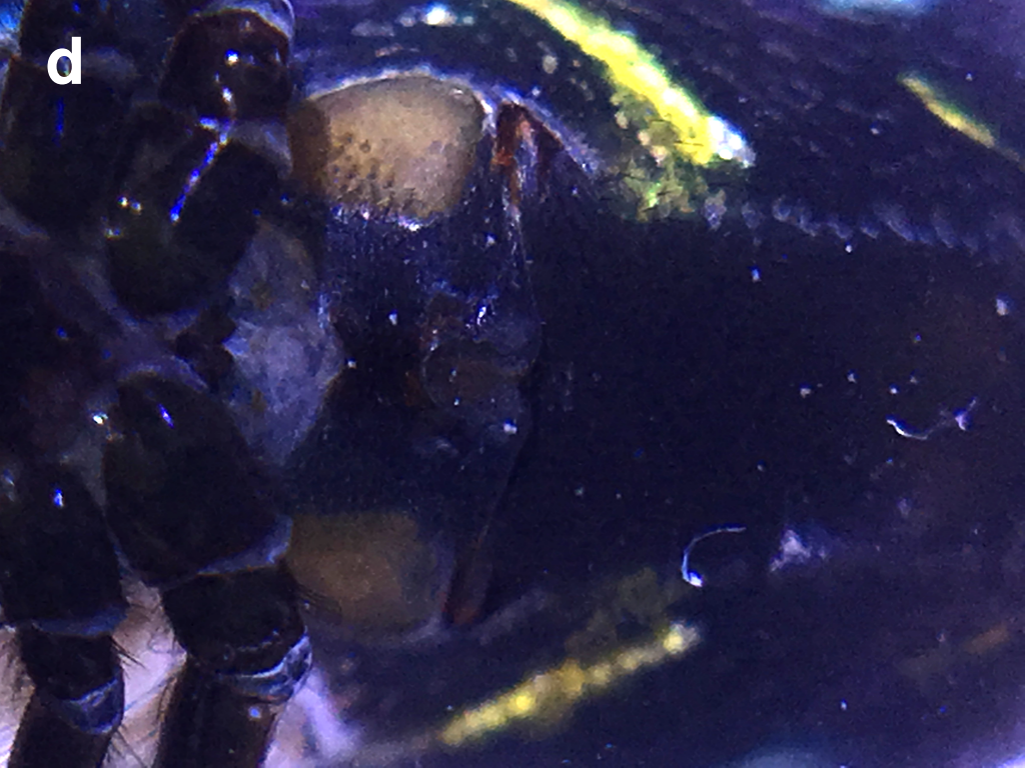
Epigynum, ventral view.

**Figure 8a.**
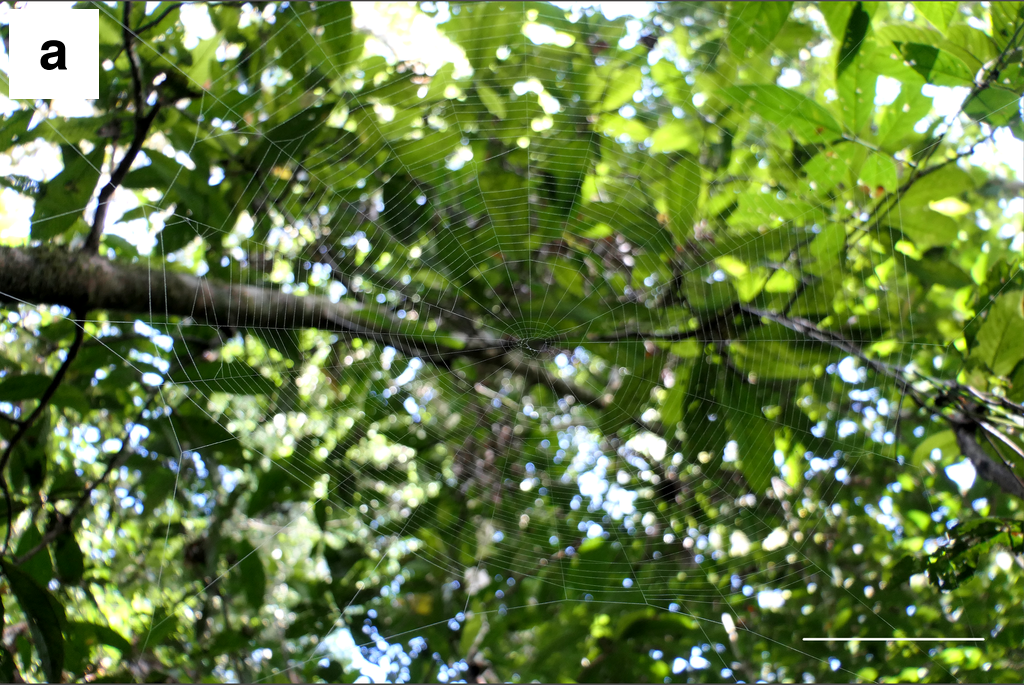
View from below. Scale bar 10 cm.

**Figure 8b.**
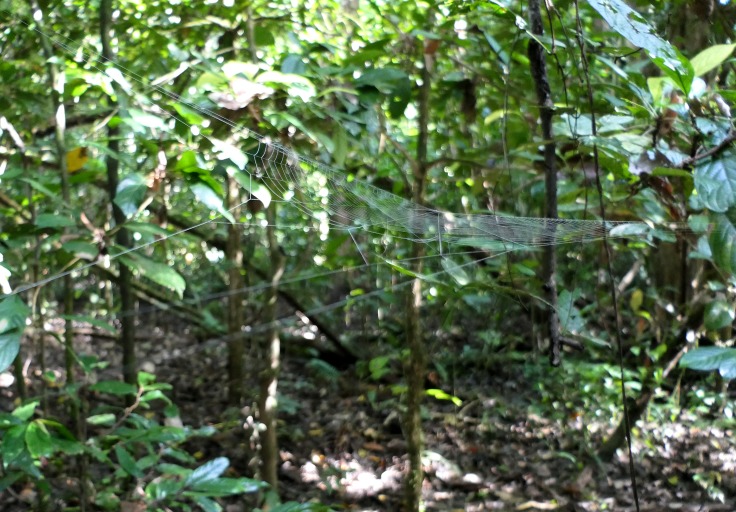
View from side.

**Table 1.** Characteristics of *Opadometa sarawakensis* webs and their habitats.

	**Specimen**	
**Web and forest characteristics**	DGFCW2018022402	DGFCW2018022611
Diameter (cm)	29	35.7
Number of radii	28	22
Number of spirals	46	33
Angle above hub	55	25
Angle below hub	35	5
Hub height from ground (cm)	150	153
Stem count (3 m radius)	35	29
Percent canopy cover	91	94
